# Supplementary material for: A Novel Handrub Tablet Loaded with Pre- and Post-Biotic Solid Lipid Nanoparticles Combining Virucidal Activity and Maintenance of the Skin Barrier and Microbiome
Source: Pharmaceutics. 2023 Dec 17;15(12):2793. doi: 10.3390/pharmaceutics15122793 (PMC10747770; doi:10.3390/pharmaceutics15122793)
Supplement: Supplementary file 1 [file pharmaceutics-15-02793-s001.zip › pharmaceutics-2717733-supplementary.pdf]

Supplementary Material

Table S1 – Table of Analysis of Variance for tablet base formulations DoE planning.

	Dependent variables p-values					
Source	Weight	Disintegra-tion time	Swelling	Height	Diameter	Hardness
Model	0.019	0.001	0.009	0.000	0.891	0.110
Linear	0.007	0.000	0.004	0.000	0.783	0.093
Diluent prop. (MCC %w/w)	0.002	0.000	0.009	0.000	0.409	0.067
[Disintegrant] (%w/w)	0.035	0.076	0.009	0.025	0.767	0.223
Disintegrant prop. (SCC %w/W)	0.148	0.001	0.003	0.007	0.795	0.079
Square	0.793	0.016	0.015	0.052	0.605	0.108
Diluent prop. (MCC %w/w)*Diluent prop. (MCC %w/w)	0.793	0.016	0.015	0.052	0.605	0.108
2-Way Interaction	0.432	0.012	0.051	0.070	0.815	0.145
Diluent prop. (MCC %w/w)*[Disintegrant] (%w/w)	0.582	0.030	0.558	0.066	0.870	1.000
Diluent prop. (MCC %w/w)*Disintegrant prop. (SCC %w/W)	0.597	0.004	0.017	0.066	0.438	0.356
[Disintegrant] (%w/w)*Disintegrant prop. (SCC %w/W)	0.182	0.546	0.144	0.102	0.749	0.047
Error						
Lack-of-Fit	0.640	0.428	0.088	1.000	0.889	*
Pure Error	-	-	-	-	-	-
Total	-	-	-	-	-	-
Model Summary						
S	0.00064	2.259	34.943	0.01414	0.17830	1.0614
R-sq	97.60%	99.70%	98.58%	99.82%	44.31%	91.93%
R-sq(adj)	92.00%	98.99%	95.25%	99.40%	0.00%	73.10%
R-sq(pred)	75.37%	93.18%	23.59%	99.60%	0.00%	0.00%

Figure S1 – Pareto charts of the standardized effects for response surface method from tablet base formulations DoE planning.

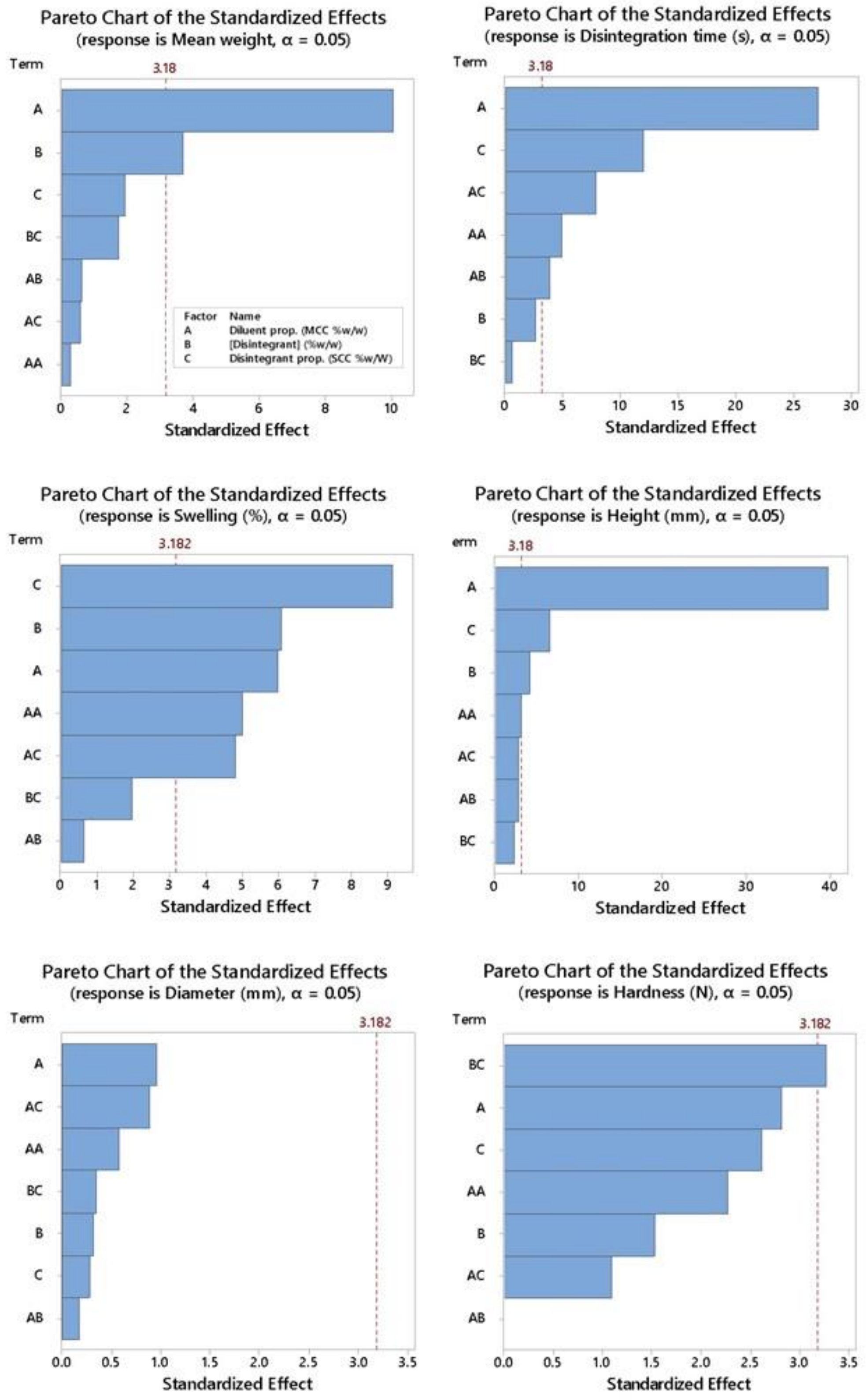

Figure S2 – Contour plots for response surface method considering parameters with significance in the analysis of variance from tablet base formulations DoE planning.

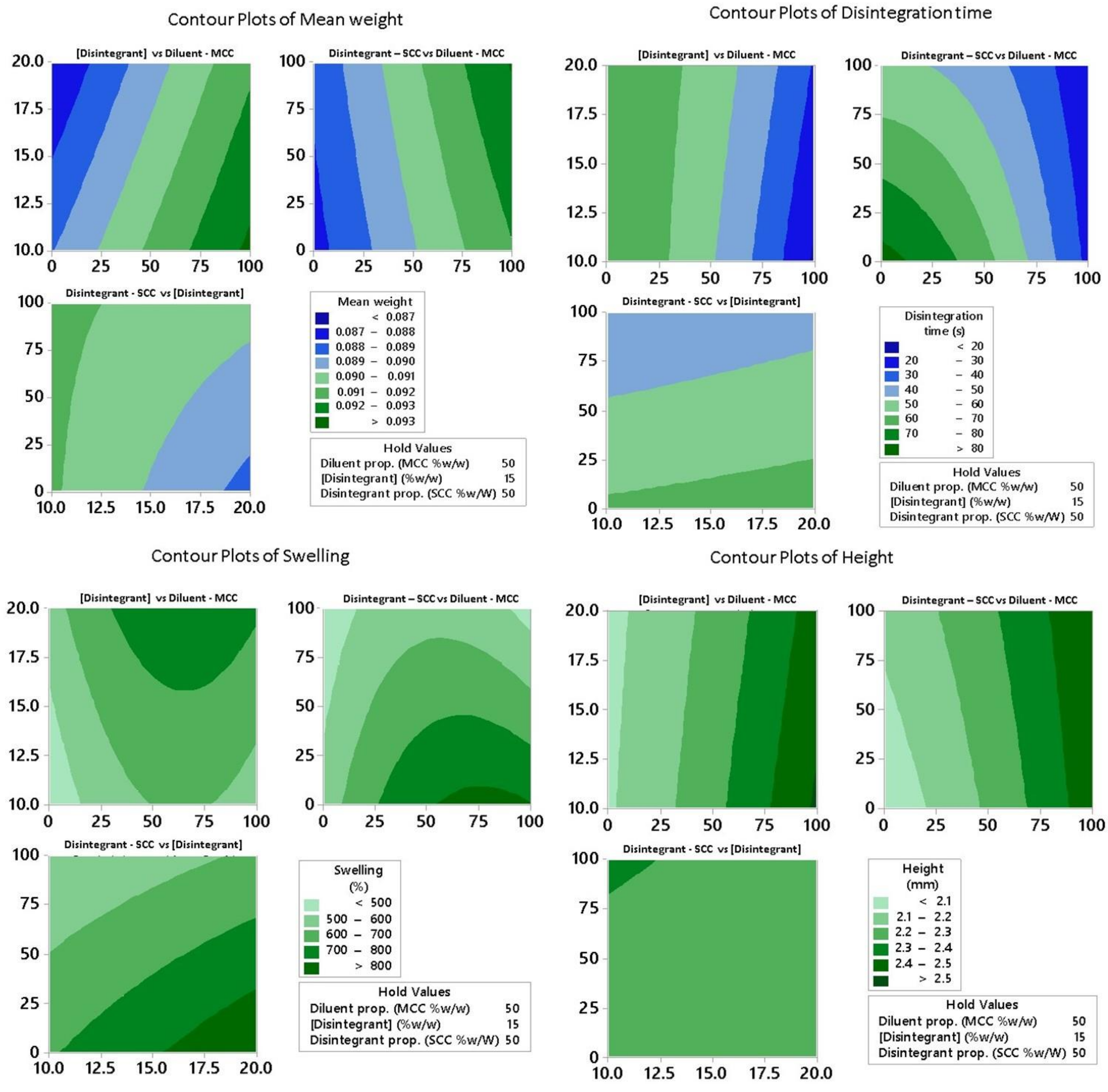

Table S2 - Response optimization from tablet base formulation DoE planning

Response	Goal	Lower	Target	Upper	Weight	Importance
Height (mm)	Maximum	2.030	2.543		1	1
Swelling (%)	Minimum		369.8	931.1	1	1
Disintegration time (s)	Minimum		16.0	85.7	1	1
Mean weight	Maximum	0.086	0.094		1	1

Solutions

Solution	Diluent Proportion (MCC%w/w)	[Disintegrant] (%w/w)	Disintegrant Proportion (SCC%w/W)	Height (mm) Fit	Swelling (%) Fit	Disintegration time (s) Fit	Mean Weight Fit	Composite Desirability
1	100.0	10	100	2.54	401.6	15.2	0.0934	0.981
2	92.8	10	100	2.51	432.2	19.9	0.0931	0.926
3	91.9	10	100	2.50	435.6	20.4	0.0930	0.919
4	89.9	10	100	2.49	443.1	21.7	0.0923	0.903

Multiple Response Prediction

Variable	Setting
Diluent prop. (MCC %w/w)	100
[Disintegrant] (%w/w)	10
Disintegrant prop. (SCC %w/W)	100

Response	Fit	SE Fit	95% CI	95% PI
Height (mm)	2.5433	0.0132	(2.5012, 2.5854)	(2.4817, 2.6050)
Swelling (%)	401.6	32.7	(297.5, 505.6)	(249.3, 553.8)
Disintegration time (s)	15.21	2.11	(8.48, 21.93)	(5.36, 25.05)
Mean weight	0.093370	0.000599	(0.091462, 0.095277)	(0.090578, 0.096162)

Figure S3 - Response optimization from tablet base formulation DoE planning

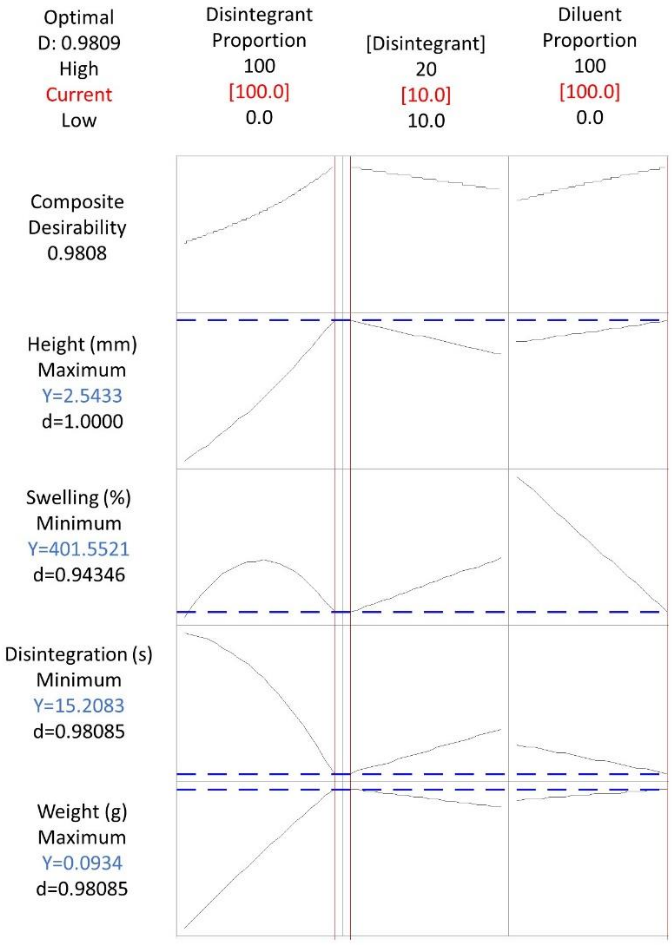

Optimal
D: 0.9809
High
Current
Low
Disintegrant Proportion
100
[100.0]
0.0
[Disintegrant]
20
[10.0]
10.0
Diluent Proportion
100
[100.0]
0.0
Composite Desirability
0.9808
Height (mm)
Maximum
Y=2.5433
d=1.0000
Swelling (%)
Minimum
Y=401.5521
d=0.94346
Disintegration (s)
Minimum
Y=15.2083
d=0.98085
Weight (g)
Maximum
Y=0.0934
d=0.98085
